# A microbial consortium alters intestinal *Pseudomonadota* and antimicrobial resistance genes in individuals with recurrent *Clostridioides difficile* infection

**DOI:** 10.1128/mbio.03482-22

**Published:** 2023-07-05

**Authors:** Ashley M. Rooney, Kyla Cochrane, Stephanie Fedsin, Samantha Yao, Shaista Anwer, Satyender Dehmiwal, Susy Hota, Susan Poutanen, Emma Allen-Vercoe, Bryan Coburn, MTOP Investigators

**Affiliations:** 1 Department of Laboratory Medicine and Pathobiology, University of Toronto, Toronto, Canada; 2 Toronto General Hospital Research Institute, University Health Network, Toronto, Canada; 3 NuBiyota, University of Guelph, Guelph, Canada; 4 Department of Microbiology, Sinai Health, Toronto, Canada; 5 Division of Infectious Diseases, University Health Network, Toronto, Canada; 6 Infection Prevention and Control Department, University Health Network, Toronto, Canada; 7 Department of Medicine, University of Toronto, Toronto, Canada; Rutgers University, Piscataway, New Jersey, USA

**Keywords:** gut microbiome, microbial consortium, fecal microbiota transplant, antibiotic resistance, anaerobes, *Proteobacteria*, *Pseudomonadota*

## Abstract

Intestinal colonization with pathogens and antimicrobial-resistant organisms (AROs) is associated with increased risk of infection. Fecal microbiota transplant (FMT) has successfully been used to cure recurrent *Clostridioides difficile* infection (rCDI) and to decolonize intestinal AROs. However, FMT has significant practical barriers to safe and broad implementation. Microbial consortia represent a novel strategy for ARO and pathogen decolonization, with practical and safety advantages over FMT. We undertook an investigator-initiated analysis of stool samples collected from previous interventional studies of a microbial consortium, microbial ecosystem therapeutic (MET-2), and FMT for rCDI before and after treatment. Our aim was to assess whether MET-2 was associated with decreased *Pseudomonadota (Proteobacteria*) and antimicrobial resistance gene (ARG) burden with similar effects to FMT. Participants were selected for inclusion if baseline stool had *Pseudomonadota* relative abundance ≥10%. Pre- and post-treatment *Pseudomonadota* relative abundance, total ARGs, and obligate anaerobe and butyrate-producer relative abundances were determined by shotgun metagenomic sequencing. MET-2 administration had similar effects to FMT on microbiome outcomes. The median *Pseudomonadota* relative abundance decreased by four logs after MET-2 treatment, a greater decrease than that observed after FMT. Total ARGs decreased, while beneficial obligate anaerobe and butyrate-producer relative abundances increased. The observed microbiome response remained stable over 4 months post-administration for all outcomes.

**IMPORTANCE:**

Overgrowth of intestinal pathogens and AROs is associated with increased risk of infection. With the rise in antimicrobial resistance, new therapeutic strategies that decrease pathogen and ARO colonization in the gut are needed. We evaluated whether a microbial consortium had similar effects to FMT on *Pseudomonadota* abundances and ARGs as well as obligate anaerobes and beneficial butyrate producers in individuals with high *Pseudomonadota* relative abundance at baseline. This study provides support for a randomized, controlled clinical trial of microbial consortia (such as MET-2) for ARO decolonization and anaerobe repletion.

## INTRODUCTION

The human gut microbiome is a source of infection among hospitalized individuals (1, 2). Gastrointestinal carriage of pathogenic antimicrobial-resistant organisms (AROs) with increased abundance of *Pseudomonadota (Proteobacteria)* or *Enterococcus* and decreased abundance of obligate anaerobes and butyrate producers is associated with elevated risks of infection in these individuals (3 - 6). Gut commensal anaerobes provide colonization resistance against opportunistic pathogens and are important mediators of immune system function and regulation as well as intestinal epithelial barrier function (7 - 10). Antibiotic use, including prophylaxis and selective decontamination of the digestive tract, is effective infection prevention strategies in some populations (5, 11). However, antibiotic use is associated with toxicity, disruption of the gut microbiota, and selection for AROs (12 - 14). Alternatives to antibiotics for ARO decolonization have the potential to confer the clinical benefits of decolonization without the specific toxicities and selection for antimicrobial resistance.

Fecal microbiota transplant (FMT) is effective for the treatment of recurrent *Clostridioides difficile* infection (rCDI) and is associated with decreased incidence of bloodstream infection in this population (15, 16). The gut microbiota of individuals with rCDI is characterized by increased abundances of stool *Pseudomonadota* including disease-causing members of *Enterobacteriaceae* (5, 17 - 19). In individuals with rCDI, FMT may restore colonization resistance by increasing beneficial anaerobes and decreasing potential pathogens (20 - 22). FMT may also be a strategy for decolonizing intestinal AROs where eradication rates have ranged from 37.5% to 87.5% in mostly small observational studies lacking a placebo control (18). However, there is inconclusive evidence that FMT is associated with decreases in antimicrobial resistance genes (ARGs) (23). Two studies demonstrated a decrease in overall ARGs (20) and patient-specific ARGs (22), while another study has shown that FMT is a potential source of ARGs (21). Currently, FMT is limited by safety and scalability challenges and may be an unsustainable solution for therapeutic use on a broad scale (24, 25).

Microbial consortia, consisting of a combination of bacterial strains isolated from healthy human donors, have been developed to overcome some limitations of FMT (26 - 29). In recent human clinical studies, microbial consortia stably colonize human stool (28) and have shown to be effective for the prevention of rCDI (26, 27, 30). However, to our knowledge, there are no studies performed in humans that assess the effect microbial consortia on *Pseudomonadota* and ARG abundance.

As previous studies have demonstrated the relationship between overgrowth of gastrointestinal *Pseudomonadota,* other pathogens, and ARO carriage with infection risk (3 - 6) as well as ARG abundance and ARO colonization (22), we aimed to assess the impact of administration of a microbial consortium, microbial ecosystem therapeutic (MET-2), on members of *Pseudomonadota* and ARG abundance. In the current study, we performed shotgun metagenomic sequencing on stool collected from participants with elevated abundances of *Pseudomonadota* in a previously published trial of MET-2 for rCDI (26) and recipients of FMT for the same indication. The abundances of *Pseudomonadota*, ARGs, obligate anaerobes, and butyrate producers in pre- and post-therapy stool samples were analyzed.

## RESULTS

### Participant characteristics

Two individuals (participants 4 and 7) from the initial trial (*n* = 19) had less than 10% *Pseudomonadota* relative abundance by 16S rRNA gene sequencing and were not subject to shotgun metagenomic sequencing in the current study. Participants were further selected for inclusion if they had high baseline *Pseudomonadota* (>10% relative abundance) measured by shotgun metagenomic sequencing and administration of a single course of therapeutic (MET-2 or FMT) relative to post-treatment sampling. A total 15/17 (88%) MET-2 for rCDI trial participants and 5/8 (62%) FMT for rCDI study participants met this inclusion criterion. Participant characteristics are outlined in Supplementary Table 1. Participants in both groups initially received vancomycin, except for participant 1 in the FMT study who received fidaxomicin. The median age of the MET-2 and FMT participants was 65 years and 67 years, respectively, and female participants were more common in both groups [MET-2, 67% (10/15); FMT, 100% (5/5)]. A single FMT donor provided stool on multiple dates for preparation of FMT. Supplementary Table 2 provides stool donation dates and to which recipients the donated stool was administered. Participant 10 who received MET-2 failed initial MET-2 administration and was retreated (stool samples before and after re-treatment are not included in this study). Participant 3 of the FMT group failed the initial course of FMT and did not receive another course within the study period. For the baseline and 1 month post-intervention analyses, data from two participants from the MET-2 interventional group were excluded due to missing 1 month samples. These two participants were included for the longitudinal analyses.

### *Pseudomonadota* abundance pre- and post-intervention

**Fig 1:**
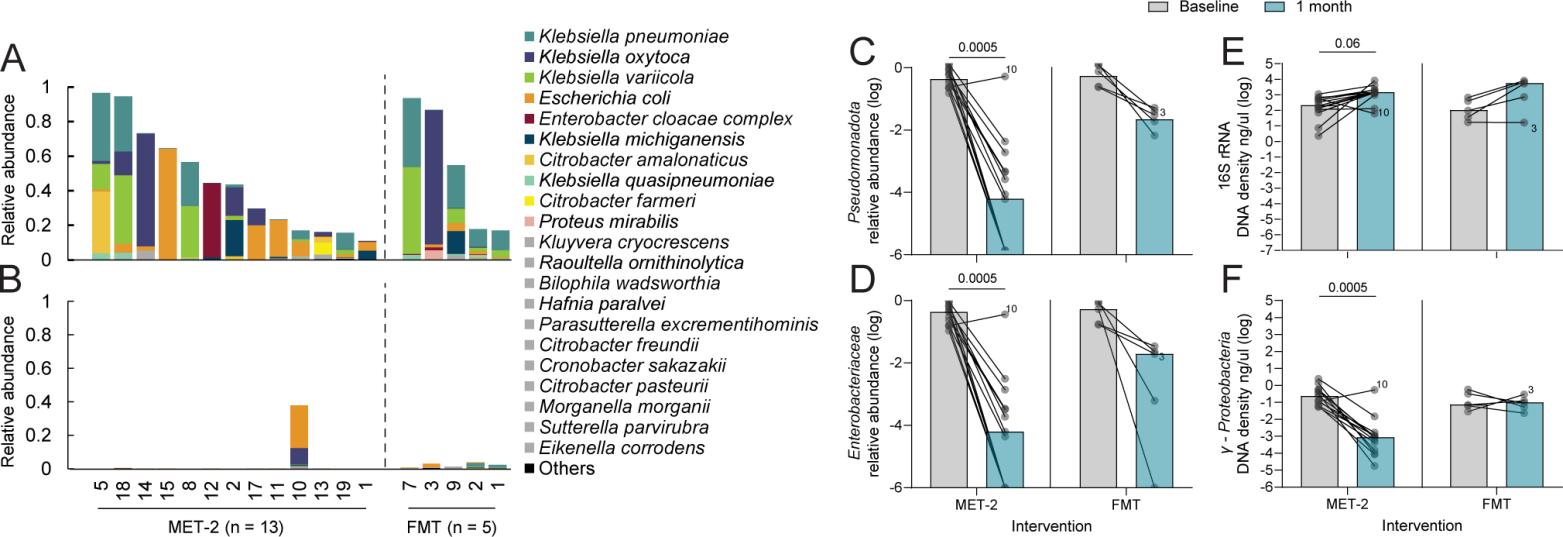
*Pseudomonadota* relative abundances in participants who received MET-2 (*n* = 13) or FMT (*n* = 5). *Pseudomonadota* are summarized at the species level in the baseline (**A**) and 1 month post-intervention stool samples (**B**). **(A and B)** Species contributing <1% relative abundance in at least one sample were aggregated as “Others.” Species contributing <5% relative abundance but ≥1% in at least one sample are in gray. (**C)** *Pseudomonadota* relative abundance between baseline and 1 month post-intervention. (**D)** *Enterobacteriaceae* relative abundance between baseline and 1 month post-intervention. (**E)** 16S rRNA DNA density (ng/μL) between baseline and 1 month post-intervention. (**F)** *γ-Proteobacteria* DNA density (ng/μL) between baseline and 1 month post-intervention. (**C–F)** Values are log-transformed, where dots represent individual participants with lines connecting the same participants measured at different time points. Participant 10 and Participant 3 are highlighted as individuals who failed initial MET-2 or FMT therapy, respectively. Medians are plotted with *P*-values displayed above the MET-2 interventional group. Pairwise analysis performed using Wilcoxon matched-pairs signed-rank test.

Analysis of the 16S rRNA gene sequencing data generated from the initial trial for all MET-2 participants (*n* = 19) (26) revealed that the median *Pseudomonadota* relative abundance decreased and remained low over time for individuals who did not require repeat treatment (*n* = 15) (Supplementary Figure 1A), while this trend was not observed in retreated individuals (*n* = 4) (Supplementary Figure 1B). Among participants with baseline and 1 month samples included in this shotgun metagenomic sequencing study, median baseline *Pseudomonadota* relative abundance for participants who received MET-2 (*n* = 13) was 44% (range, 11%–97%) and for participants who received FMT (*n* = 5) was 55% (range, 17%–93%) (Mann-Whitney *U*-test, *P* = 0.70). At baseline, the most common and abundant *Pseudomonadota* genera in the MET-2 and FMT groups were *Klebsiella,* which included *K. pneumoniae, K. oxytoca, K. variicola, K. michiganensis,* and *K. quasipneumoniae*. Other species included *Escherichia coli, Enterobacter cloacae* complex, and *Citrobacter* spp. (Fig. 1A). At 1 month post-intervention, the *Pseudomonadota* relative abundance decreased to a median relative abundance of 0.01% (range, 0%–38%) in the MET-2 group (Wilcoxon signed-rank test of baseline vs 1 month, *P* = 0.0005) and 2.2% (range, 0.5%–3.7%) in the FMT group (Fig. 1B and C) with a median log_2_ *Pseudomonadota* decrease of 11.8 and 4.9, respectively. There was a decrease in the relative abundances of γ-*Proteobacteria* (Supplementary Figure 2) and *Enterobacteriaceae* (Fig. 1D) (Wilcoxon signed-rank tests of baseline vs 1 month MET-2, *P* = 0.0005). One participant (Participant 10) failed initial MET-2 therapy for rCDI (26) and was observed in the current study to have a baseline *Pseudomonadota* relative abundance of 17% that increased to 38% by approximately 1 month post-MET-2 administration.

It is possible that MET-2 or FMT administration is displacing *Pseudomonadota* but not decreasing their density, which cannot be captured by sequencing-based relative abundance measurements. We quantified total DNA density of 16S rRNA (total bacteria) and *γ-Proteobacteria* by quantitative polymerase chain reaction (qPCR). Between baseline and 1 month post-administration, the median 16S rRNA stool density post-MET-2 or FMT increased by 1 log from 215 ng/µL (range, 2.6–1,230 ng/µL) to 1,511 ng/µL (range, 68–8,863 ng/µL) and 1.7 logs from 107 ng/µL (range, 19–737 ng/µL) to 5,628 ng/µL (range, 18–8,783 ng/µL), respectively, although the trend was not significant (Wilcoxon signed-rank test of baseline vs 1 month MET-2, *P* = 0.06) (Fig. 1E). The median *γ-Proteobacteria* density decreased by 2.5 logs from 0.2 ng/µL (range, 0.06–2.5 ng/µL) to 0.0008 ng/µL (range, 0.00002–0.5 ng/µL) post-MET-2 administration (Wilcoxon signed-rank test of baseline vs 1 month post-MET-2, *P* = 0.0005), while no change in *γ-Proteobacteria* density between baseline (median, 0.08 ng/µL; range, 0.03–0.6 ng/µL) and 1 month post-FMT administration (median, 0.1 ng/µL; range, 0.02–0.3 ng/µL) was observed (Fig. 1F). As the trend of overall bacterial density was increasing with no change in *γ-Proteobacteria* density post-FMT, we reasoned that FMT may be contributing donor-derived *Pseudomonadota*. 16S rRNA gene sequencing data were available for three donor FMT samples, and the average *Pseudomonadota* and *γ-Proteobacteria* relative abundance was 5% (range, 3%–8%) and 3% (range, 2%–5%), respectively, consistent with this hypothesis.

As a comparator to non-microbial therapies, a total of six individuals with CDI treated with vancomycin were identified from published data sets (31, 32). The baseline *Pseudomonadota* abundance was 36% (range, 0.6%–81%), where the most abundant family was *Enterobacteriaceae* (Supplementary Figure 3A). At 1 month post-vancomycin treatment, *Pseudomonadota* relative abundance was 38% (range, 14%–78%) (Supplementary Figure 3B). There was no observed decrease in *Pseudomonadota* or *Enterobacteriaceae* relative abundance between baseline and 1 month post-vancomycin stool samples (Supplementary Figure 3C and D).

### Antimicrobial resistance genes pre- and post-intervention

**Fig 2:**
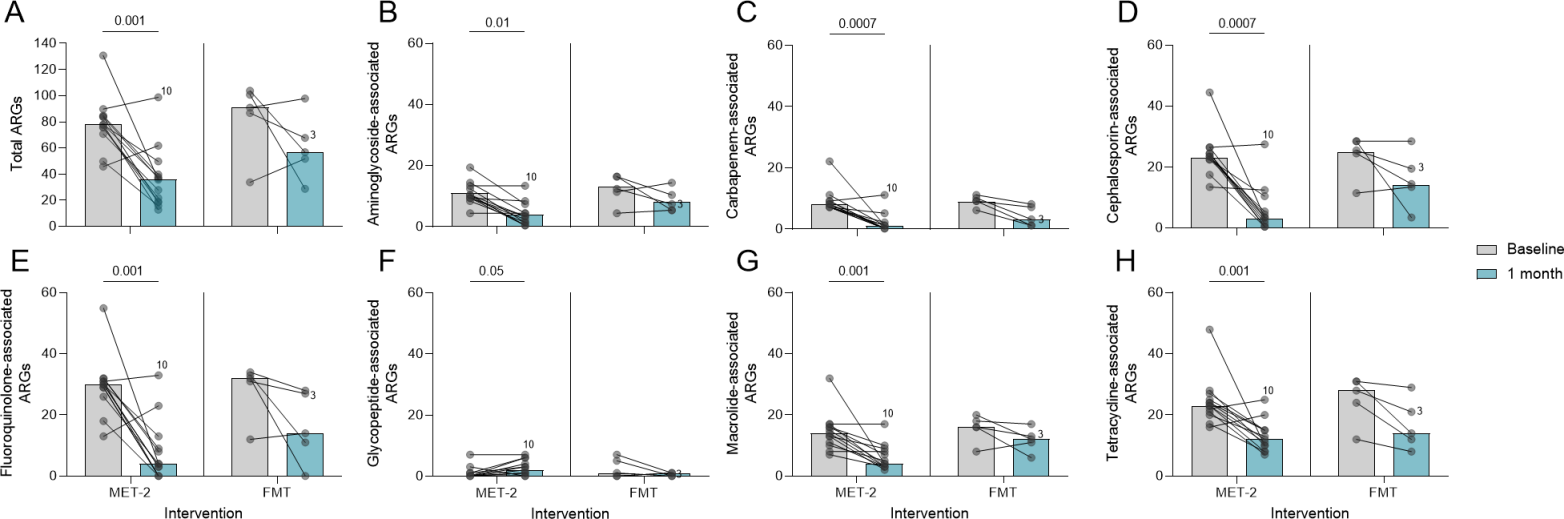
(A) Antimicrobial resistance genes (ARGs) in participants who received MET-2 (*n* = 13) or FMT (*n* = 5) between baseline and 1 month post-intervention. (**B–H)** ARGs categorized by drug class-associated resistance in participants who received MET-2 (*n* = 13) or FMT (*n* = 5) between baseline and 1 month post-intervention. Drug classes analyzed include aminoglycosides (**B**), carbapenems (**C**), cephalosporins (**D**), fluoroquinolones (**E**), glycopeptides (**F**), macrolides (**G**), and tetracyclines (**H**). Dots represent individual participants with lines connecting the same participants measured at different time points. Participant 10 and Participant 3 are highlighted as individuals who failed initial MET-2 or FMT therapy, respectively. Medians are plotted with *P*-values displayed above the MET-2 interventional group. Pairwise analysis performed using Wilcoxon matched-pairs signed-rank test. Each dot represents an individual with the baseline and 1 month time points included.

Total ARGs were measured between baseline and 1 month post-intervention. The ARG numbers at baseline were similar between interventions (Mann-Whitney *U*-test, *P* = 0.16), where the median number of ARGs for participants who received MET-2 was 78 (range, 46–131) and for participants who received FMT was 91 (range, 34–104). There was a decrease in the total number of ARGs by 1 month after the MET-2 and FMT interventions (Fig. 2A), except for two individuals in both groups where ARGs increased (Fig. 2A). There was a strong positive correlation (Spearman *r* = 0.70, *P* < 0.0001) between the total number of ARGs detected and *Pseudomonadota* relative abundance in the MET-2 interventional group (Supplementary Figure 4A), while there was a weak positive correlation (Spearman *r* = 0.36, *P* = 0.31) between the total number of ARGs detected and *Pseudomonadota* relative abundance in the FMT interventional group (Supplementary Figure 4B).

The number of ARGs categorized by drug class-associated resistance for the baseline and 1 month post-MET-2 or FMT interventions is shown in Fig. 2B through H. At baseline in both interventional groups, ARGs conferring resistance to fluoroquinolones (Fig. 2E), cephalosporins (Fig. 2D), and tetracyclines (Fig. 2H) were the most abundant. The median number of ARGs associated with resistance to the drug classes analyzed all decreased after either intervention. However, ARGs conferring resistance to glycopeptide antibiotics increased in the MET-2 interventional group (Fig. 2F). Clinically relevant vancomycin resistance genes (*vanA–vanE* and *vanG*) (33) were detected in 2/13 participants who received MET-2. In participant 10, *vanA* was detected in baseline and at 1 month post-MET-2 administration, while *vanC* was detected in participant 18 (Supplementary Figure 5A). The median *Enterococcus* relative abundance at 1 month post-MET-2 administration was 0% (range, 0%–21%), where participants 10 and 18 had an *Enterococcus* relative abundance of 21% and 0.2%, respectively, suggesting that the increase in ARGs conferring resistance to glycopeptides in the MET-2 group overall was not due to vancomycin-resistant *Enterococcus* (Supplementary Figure 5B).

High-risk ARGs (*n* = 73) outlined in the study by Zhang and colleagues (34) were quantified in the current study. In both interventional groups at baseline, the median number of high-risk ARGs was 4 (range, 1–6), which decreased by 1 month post-MET-2 to a median of 2 (range, 0–4) while no change post-FMT (Supplementary Figure 6). The ARGs *bacA*, *mdtE*, *TolC*, and *ermB* were common at baseline in the MET-2 and FMT recipients (Supplementary Figure 7 and 8) but generally not detected by 1 month post-MET-2, except for ermB which remained detected in most individuals or became detected by 1 month. The beta-lactamases *bla_CTX-M-15_*, *bla_OXA-1_*, *bla_SHV-1_*, and *bla_TEM-1_* detected at baseline were no longer detected by 1 month post-MET-2 (Supplementary Figure 7).

### Obligate anaerobes and butyrate producers pre- and post-intervention

**Fig 3:**
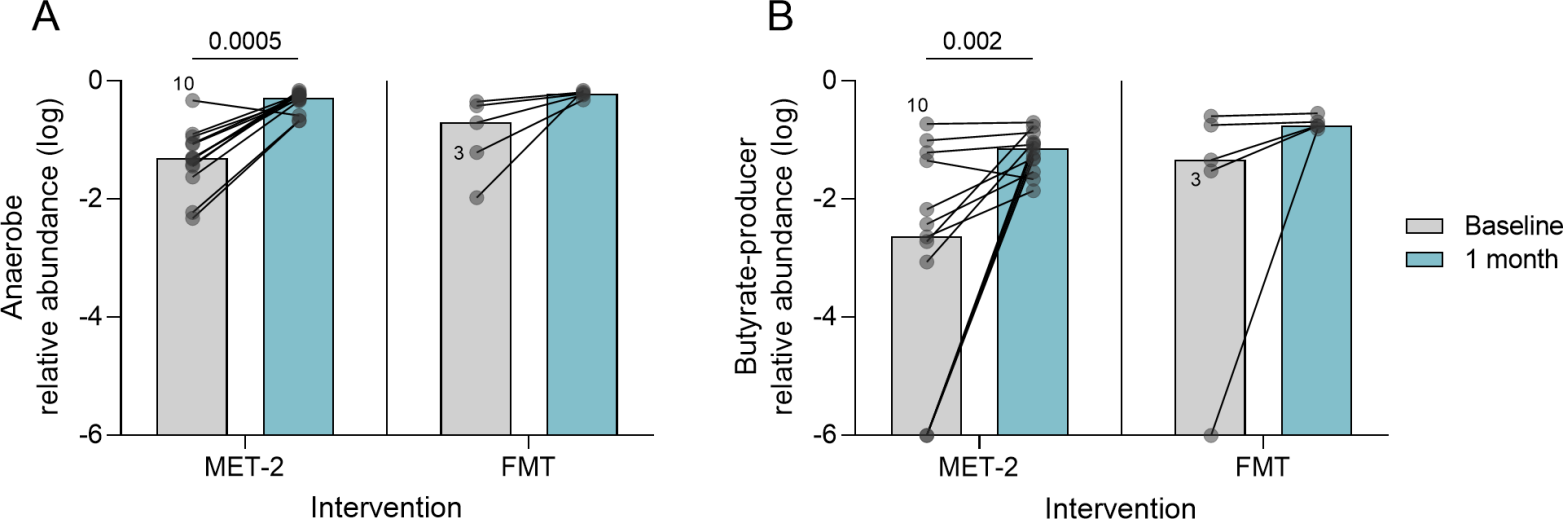
Obligate anaerobes (**A**) and butyrate-producer (**B**) log-transformed relative abundances in participants who received MET-2 (*n* = 13) or FMT (*n* = 5) between baseline and 1 month post-intervention. Dots represent individual participants with lines connecting the same participants measured at different time points. Participant 10 and Participant 3 are highlighted as individuals who failed initial MET-2 therapy. Medians are plotted with *P*-values displayed above the MET-2 interventional group. Pairwise analysis performed using Wilcoxon matched-pairs signed-rank test.

Because of their significance in ARO colonization resistance and intestinal epithelial barrier and systemic immune function, we quantified obligate anaerobes and butyrate-producer relative abundances. At baseline, the median obligate anaerobe relative abundances for the MET-2 and FMT interventional groups were 5% (range, 0.5%–47%) and 20% (range, 1.1%–45%), respectively (Mann-Whitney *U*-test, *P* = 0.21). The median butyrate-producer relative abundances for MET-2 and FMT interventional groups were 0.002% (range, 0%–0.2%) and 0.05% (range, 0%–0.3%), respectively (Mann-Whitney *U*-test, *P* = 0.20). There was an observed increase in obligate anaerobe (Fig. 3A) (MET-2; *P* = 0.0005) and butyrate-producer (Fig. 3B) (MET-2; *P* = 0.002) relative abundances between baseline and 1 month post-MET-2 and FMT samples.

### Microbiome response over time

**Fig 4:**
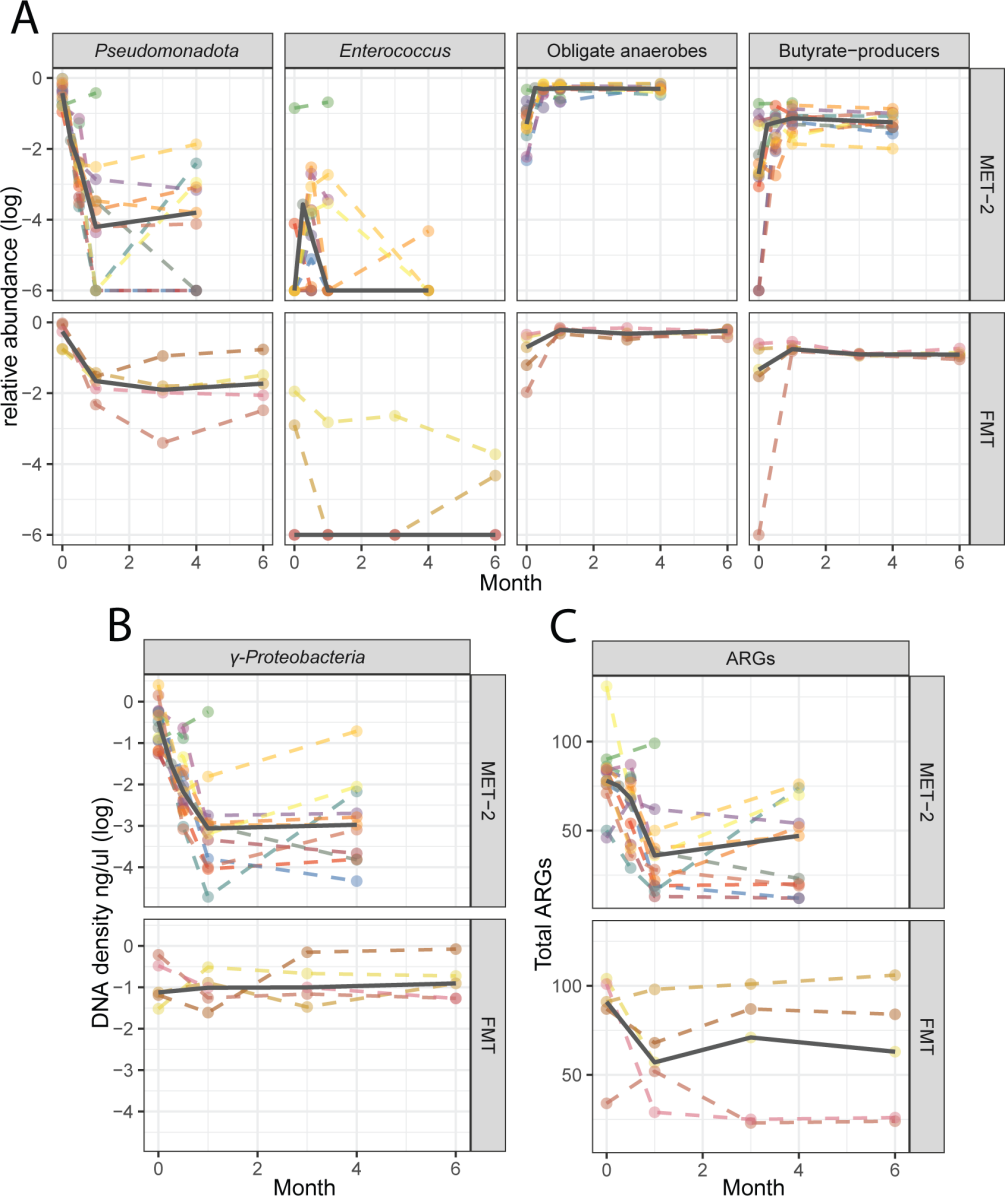
(A) Relative abundances of *Pseudomonadota, Enterococcus*, obligate anaerobes, and butyrate producer. (**B)** *γ-Proteobacteria* DNA density (ng/μL) and (**C**) total antimicrobial resistance genes (ARGs) in participants who received MET-2 (*n* = 15) or FMT (*n* = 5) measured over time in months. (**A and B)** Values are log transformed. Dots represent individual participants with dashed lines connecting the same participants measured at different time points. The solid line represents the median across time points.

To assess stability of the treatment-associated changes in microbiome composition, *Pseudomonadota*, *Enterococcus,* obligate anaerobe, and butyrate-producer relative abundances (Fig. 4A), as well as *γ-Proteobacteria* absolute abundance (Fig. 4B) and total ARGs (Fig. 4C), were assessed at baseline, 0.5, 1, and 4 months or baseline, 1, 3, and 6 months post-intervention for MET-2 (*n* = 15) and FMT (*n* = 5), respectively. The medians of all measured outcomes remained lower than baseline and stable at the final sampling timepoints for both interventions.

## DISCUSSION

In this analysis of adults with rCDI colonized with *Pseudomonadota (Proteobacteria*), we observed that administration of a microbial consortium (MET-2) was associated with decreased *Pseudomonadota* and ARGs. The observed changes were similar or greater in magnitude to the effect of FMT for these outcomes. Obligate anaerobe and butyrate-producer relative abundances by 1 month post-intervention increased in a similar manner to FMT. The *γ-Proteobacteria* absolute abundance, measured by qPCR, decreased in the microbial consortium interventional group along with non-significant increases in 16S rRNA density. In the FMT group, there was a trend toward increased 16S rRNA density with no change in *γ-Proteobacteria* density by 1 month post-administration, possibly due to transfer of donor-derived *Pseudomonadota*. In an exploratory longitudinal analysis, we observed that all microbiome outcomes, including *Enterococcus* relative abundance observed at 1 month, remained stable at 4 and 6 months after administration of the microbial consortium and FMT, respectively. Overall, our findings indicate that microbial consortium administration produces effects consistent with FMT.

To our knowledge, this is the first study to assess the effects of a therapeutic microbial consortium on *Pseudomonadota* and ARGs in the human gut microbiome. The results observed after MET-2 administration were similar to those after FMT administration in this study as well as to published studies using shotgun metagenomic sequencing to assess the effects of FMT on the gut microbiota composition of patients with rCDI. In published studies, multiple-log decreases in *Pseudomonadota* relative abundance have been documented, along with increases in anaerobic *Bacillota (Firmicutes*) and *Bacteroidota (Bacteroidetes)* (20 - 22).

We observed that MET-2 was associated with a decrease in ARGs by 1 month which is similar to published reports using FMT for this indication (20, 22, 35). In our analysis, we did not observe ARG decreases in the five participants with rCDI who received FMT. We did not perform shotgun metagenomic sequencing on the donor material from the FMT group, so could not ascertain if the ARGs are being introduced by the FMT. However, reports of the effects of FMT on ARG numbers have been heterogeneous (23). In previous studies, even after extensive screening of donor stool, FMT administration was the source of an antibiotic-resistant *E. coli* bacteremia (24), while Leung and colleagues (21) have reported that FMT may be a source of clinically relevant ARGs.

Our study has multiple limitations. This was not a randomized comparison of MET-2 and FMT, but a *post hoc* analysis of participants treated for rCDI. This study was not designed to determine if one strategy is superior to another; thus, it would be inappropriate to make direct comparisons between the effects of MET-2 and FMT on our measured outcomes. We aimed to instead use the FMT group to calibrate the microbiome effects observed post-MET-2 administration. There is no control group to account for spontaneous decolonization of *Pseudomonadota* and ARGs. However, our analyses of published data sets of patients with CDI pre- and post-vancomycin therapy suggest that it is possible that *Pseudomonadota* can remain colonized or increase in some cases 1 month after vancomycin. Others have also reported persistence of ARGs in the gut microbiome acquired post-antibiotic therapy for up to 2 years (36). Our results were of the metagenome only. The measures of *Pseudomonadota* abundance do not indicate pathogenicity as these organisms are commensals of the gut and are carried in healthy individuals. ARGs do not indicate the presence of AROs as genotype does not necessarily indicate phenotype. However, increased abundances of *Pseudomonadota* as well as *Enterococcus* in the gut microbiome are associated with increased risk of infection (4 - 6). However, others have reported associations between ARG burden and ARO colonization (22), where ARO colonization places individuals at increased risk of infection with these organisms (3). Although we observed a multiple-log decrease in *Pseudomonadota* and ARGs, these results are limited by sequencing depth (37) and may not be associated with complete decolonization of the gastrointestinal tract or host. Lastly, we did not link microbiome changes to any clinical outcomes. Relative abundances of 20%–30% have been previously associated with risks such as bacteremia in allogeneic hematopoietic cell transplant patients (5) and patients in a long-term acute care hospital (38); it is uncertain whether the larger decreases in *Pseudomonadota* and ARG abundance observed in the MET-2 recipients are associated with additional benefit over simply decreasing abundance below this (or another) risk-associated threshold.

In conclusion, administration of a microbial consortium for rCDI in participants colonized with a high relative abundance of potential pathogens has similar effects to FMT for decreasing intestinal *Pseudomonadota* and ARGs, while increasing obligate anaerobes and beneficial butyrate producers. Given the practical limitations and potential safety concerns of FMT, a trial of microbial consortia for pathogen and ARG decolonization is warranted.

## MATERIALS AND METHODS

### Study design and participants

This is a study of participants 18 years or older with rCDI, defined as one or more recurrences of CDI, who participated in separate prospective cohorts evaluating the effects of either a microbial consortium (MET-2), which has been previously described (26), or FMT (S.H, S.P, S.F, S.D, S.A., unpublished data) on rCDI recurrence. As part of the Microbiota Therapeutics Outcomes Program, the FMT for rCDI study is currently ongoing in Toronto, Ontario, Canada. Donor screening protocol, the preparation of the FMT, and participant inclusion and exclusion criteria are outlined in Supplementary Methods 1.

Briefly, both cohorts were on antibiotic therapies prior to the therapeutic intervention. Participants who received FMT underwent bowel preparation prior to FMT. FMT was administered via enema three times over the course of 7 days. Participants receiving MET-2 did not receive bowel preparation prior to the intervention. Initially, 10 MET-2 capsules were taken orally for 2 days, and then three capsules were taken orally for 8 days. In the current study, we selected individuals from either cohort with ≥10% *Pseudomonadota* relative abundance in the baseline stool sample based on 16S rRNA gene and shotgun metagenomic sequencing and did not receive additional MET-2 or FMT within a month after initial administration. This study had research ethics approval.

### Sample collection and processing

Stool sample collection and 16S sequencing from the MET-2 trial was described previously (26). Briefly, we analyzed the stool samples collected at baseline prior to MET-2, as well as approximately 2 weeks, 1 month, and 4 months post-MET-2. For the FMT study, stool samples were collected from recipients at baseline prior to FMT, as well as 1, 3, and 6 months post-FMT. All stool samples were stored at −80°C until further use. Stool samples (0.25 g) were subject to DNA extraction using the DNeasy PowerSoil Pro Kit (Qiagen, Carlsbad, CA, USA) and stored at −20°C. DNA concentration was measured using the Qubit Fluorometer (Thermo Fisher, Waltham, MA, USA) following the manufacturer’s instructions. Prior to library preparation, DNA was diluted to approximately 100 ng in DNase/RNase free water. Sequencing libraries were generated using the DNA Prep Kit (Illumina, San Diego, CA, USA) and the IDT for Illumina UD Indexes (Illumina). Libraries were stored at −20°C. Libraries were manually pooled and sequenced at 2 × 150 bp using the SP flowcell on the NovaSeq 6000 at the Princess Margaret Genomics Centre. Donor FMT from three separate samples were subject to 2 × 150 bp 16S rRNA sequencing of the V4 region using a universal forward primer and uniquely barcoded reverse primer for multiplexing (39). For amplification of the 16S amplicon, the reaction mixture contained 12.5 μL of KAPA2G Robust HotStart ReadyMix (KAPA Biosystems, Wilmington, MA, USA), 1.5 μL of 10 μm forward and reverse primers, 7.5 μL of sterile water, and 2 μL of DNA. The cycling conditions were 95**°**C for 3 minutes, 18 cycles at 95**°**C for 15 seconds, 50**°**C for 15 seconds, and 72**°**C for 15 seconds, followed by 5 minutes at 72**°**C. All reactions were performed in triplicate, assessed on a 1% TBE gel, and pooled to reduce amplification bias. Pooled triplicates were quantified using PicoGreen and combined by even concentrations. The library was then purified using Ampure XP beads and loaded onto the Illumina MiSeq at the Centre for the Analysis of Genome Evolution and Function at the University of Toronto.

### Outcomes

The primary outcome was change in *Pseudomonadota* relative abundance between baseline and approximately 1 month (30 days ± 10 days) post-intervention (MET-2 and FMT). The secondary outcomes included total ARGs as well as obligate anaerobe and butyrate-producer relative abundance between baseline and 1 month post-intervention (MET-2 and FMT). Lastly, exploratory longitudinal analyses of *Pseudomonadota*, *Enterococcus,* obligate anaerobe, and butyrate-producer relative abundances, as well as total ARGs and absolute abundance of *γ-Proteobacteria* up to 4 months in the MET-2 interventional group and 6 months in the FMT interventional group, were performed.

### Sequence data processing

Sequence quality was assessed with FastQC v0.11.9 (40). As the quality was high, no sequence trimming was performed. Nextera adapters were trimmed with Trimmomatic v0.39 (41). Human and phiX reads were removed with KneadData v0.7.2 (42). Taxa were identified from quality-processed reads using Metaphlan3 v3.0.13 (43). To ensure an even sampling depth for ARG detection, quality processed reads were subsampled to 12,328,297 reads, which represents the lowest sequencing depth that retained all baseline and 1 month samples from the MET-2 and FMT interventional groups. Based on the performance characteristics of shotgun sequencing metagenomic samples for the detection of ARGs (37), 12,328,297 reads per sample provides a detection frequency ≥90% for all ARGs to relative abundances of ≥3%. The subsampled reads were assembled into contigs using metaSPades v3.15.3 (44) with the recommended k-mer lengths of 21, 33, 55, and 77. To predict the ARGs from metagenome-assembled contigs, RGI *main* v5.1.0 of the CARD (45) was used on default settings (perfect and strict hits identified only), specifying DIAMOND v0.8.36 (46) as the local aligner and the *–low_quality* flag. RGI’s *heatmap* v5.1.0 function was used to categorize ARGs based on drug class-associated resistance. ARGs were considered high risk based on the list of high-risk ARGs (rank 1) published previously (34). The UNOISE pipeline, available through USEARCH v11.0.667 and VSEARCH v2.10.4, was used for 16S rRNA sequence analysis for the three donor FMT samples (47 - 50). Taxonomy assignment was executed using SINTAX, available through USEARCH, and the UNOISE compatible Ribosomal Database Project database v16, with a minimum confidence cutoff of 0.8 (51). Published 16S rRNA sequences of stool samples from vancomycin-treated patients with CDI pre-treatment and approximately 1 month post-treatment were analyzed to measure *Pseudomonadota* relative abundance, as a reference (31, 32), using QIMME2 v2022.8 (52). Processed 16S rRNA sequencing data of stool samples collected from the initial MET-2 trial was analyzed for *Pseudomonadota* relative abundance. 16S rRNA sequencing and data processing was described previously (26).

### Total bacterial and *γ-proteobacteria* qPCR for absolute abundance quantification

Total bacterial DNA was measured by targeting the 16S rRNA gene in each fecal sample and DNA extraction negative controls with the forward primer (5′-TCCTACGGGAGGCAGCAGT-3′), the reverse primer (5′-GGACTACCAGGGTATCTAATCCTGTT-3′), and probe (FAM-5′-CGTATTACCGCGGCTGCTGGCAC-3′-NFQMGB) (Applied Biosystems, Waltham, MA, USA). Density of *γ-Proteobacteria* in each fecal sample and DNA extraction negative controls were measured using qPCR with the forward primer (5′-TCGTCAGCTCGTGTYGTGA-3′), the reverse primer (5′-CGTAAGGGCCATGATG-3′) (53), and probe (HEX-5′-AACGAGCGC-ZEN-AACCCTTWTCCY-3′-FQ-IABk) (Integrated DNA Technologies, Coralville, IA, USA). A standard curve of *E. coli* DNA was amplified with each qPCR reaction. *E. coli* DNA concentration (ng/μL) was measured using the Qubit Fluorometer (Thermo Fisher) following the manufacturer’s instructions. The target DNA density in each sample was estimated based on the *E. coli* standard curve. For the 16S rRNA and *γ-Proteobacteria* qPCR reactions, the limit of detection was approximately 10^−7^ and 10^−6^ ng/μL, respectively, where a qPCR cycle threshold of ≥40 was considered no detectable target DNA.

### Microbiota analyses and anaerobe classification

*Pseudomonadota* content in each sample was summarized at the species level, and overall relative abundance was quantified at the phylum level. *Enterobacteriaceae, γ-Proteobacteria,* and *Enterococcus* relative abundances were also quantified. Obligate anaerobe and butyrate-producer diversity in each sample was summarized and relative abundance determined at the species level.

We used *Bergey’s Manual of Systematic Bacteriology* volumes 2–5 to manually classify species-level taxa as obligate anaerobes and butyrate producers based on descriptors in the manuals such as “strictly anaerobic,” “anaerobic,” or “obligate anaerobe” as well as “produces butyrate,” “forms butyrate,” or “butyric acid is an end product” (54 - 58). If the manual did not have descriptive terms for butyrate production, taxa were not considered butyrate producers.

Microbiome measurements were assessed at baseline, 30 days (± 10 days) and up to 4 or 6 months post-intervention for MET-2 and FMT recipients, respectively.

### Statistical analyses

Samples were grouped by intervention received (MET-2 or FMT) and stratified by timepoint. To compare relative abundances between baseline and 1 month post-intervention samples, 0.000001 was added to relative abundances for all taxa (to account for zeros), then log transformed. Pairwise analyses were performed using the non-parametric Wilcoxon matched-pairs signed-rank test on log-transformed relative abundances, DNA density, and total number of ARGs within interventional groups between the baseline and 1 month post-intervention timepoints. The non-parametric Mann-Whitney *U*-test was used to compare groups. The non-parametric Spearman’s correlation was performed to test the relationship between total ARGs and *Pseudomonadota* abundance. Statistical analyses were performed in GraphPad Prism v9.0.3. Statistical analyses were not performed between timepoints for the individuals receiving FMT.

### Role of the microbial consortium manufacturer

NuBiyota is the manufacturer of the microbial consortium (MET-2) assessed in this study. The company funded the original MET-2 trial (26), but did not provide additional funding for the current study. In the current study, the company was not involved in the study design, analysis and interpretation, or manuscript preparation.

## Data Availability

Processed shotgun metagenomic data (adapter-trimmed and human and phiX reads removed) were deposited at the European Nucleotide Archive under the accession number PRJEB56674.

## SUPPLEMENTAL MATERIAL

Supplemental Figures mbio.03482-22-s0001.docx Figures S1 to S8.

Supplemental Methods mbio.03482-22-s0002.docx FMT donor screening and stool preparation.

Table S1 mbio.03482-22-s0003.docx Participant characteristics.

Table S2 mbio.03482-22-s0004.docx Donor stool collection and FMT administration dates.

ASM does not own the copyrights to Supplemental Material that may be linked to, or accessed through, an article. The authors have granted ASM a non-exclusive, world-wide license to publish the Supplemental Material files. Please contact the corresponding author directly for reuse.

## References

[1] Taur Y , Pamer EG . 2013. The intestinal microbiota and susceptibility to infection in immunocompromised patients. Curr Opin Infect Dis 26:332–337. doi:10.1097/QCO.0b013e3283630dd3 23806896 PMC4485384

[2] Freedberg DE , Zhou MJ , Cohen ME , Annavajhala MK , Khan S , Moscoso DI , Brooks C , Whittier S , Chong DH , Uhlemann A-C , Abrams JA . 2018. Pathogen colonization of the gastrointestinal microbiome at intensive care unit admission and risk for subsequent death or infection. Intensive Care Med 44:1203–1211. doi:10.1007/s00134-018-5268-8 29936583 PMC6309661

[3] McConville TH , Sullivan SB , Gomez-Simmonds A , Whittier S , Uhlemann A-C . 2017. Carbapenem-resistant Enterobacteriaceae Colonization (CRE) and subsequent risk of infection and 90-day mortality in critically ill patients, an observational study. PLoS One 12:e0186195. doi:10.1371/journal.pone.0186195 29023567 PMC5638409

[4] Magruder M , Sholi AN , Gong C , Zhang L , Edusei E , Huang J , Albakry S , Satlin MJ , Westblade LF , Crawford C , Dadhania DM , Lubetzky M , Taur Y , Littman E , Ling L , Burnham P , De Vlaminck I , Pamer E , Suthanthiran M , Lee JR . 2019. Gut uropathogen abundance is a risk factor for development of bacteriuria and urinary tract infection. Nat Commun 10:5521. doi:10.1038/s41467-019-13467-w 31797927 PMC6893017

[5] Stoma I , Littmann ER , Peled JU , Giralt S , van den Brink MRM , Pamer EG , Taur Y . 2021. Compositional flux within the intestinal microbiota and risk for bloodstream infection with gram-negative bacteria. Clin Infect Dis 73:e4627–e4635. doi:10.1093/cid/ciaa068 31976518 PMC8662789

[6] Taur Y , Xavier JB , Lipuma L , Ubeda C , Goldberg J , Gobourne A , Lee YJ , Dubin KA , Socci ND , Viale A , Perales M-A , Jenq RR , van den Brink MRM , Pamer EG . 2012. Intestinal domination and the risk of bacteremia in patients undergoing allogeneic hematopoietic stem cell transplantation. Clin Infect Dis 55:905–914. doi:10.1093/cid/cis580 22718773 PMC3657523

[7] Kelly CJ , Zheng L , Campbell EL , Saeedi B , Scholz CC , Bayless AJ , Wilson KE , Glover LE , Kominsky DJ , Magnuson A , Weir TL , Ehrentraut SF , Pickel C , Kuhn KA , Lanis JM , Nguyen V , Taylor CT , Colgan SP . 2015. Crosstalk between microbiota-derived short-chain fatty acids and intestinal epithelial HIF augments tissue barrier function. Cell Host Microbe 17:662–671. doi:10.1016/j.chom.2015.03.005 25865369 PMC4433427

[8] Byndloss MX , Olsan EE , Rivera-Chávez F , Tiffany CR , Cevallos SA , Lokken KL , Torres TP , Byndloss AJ , Faber F , Gao Y , Litvak Y , Lopez CA , Xu G , Napoli E , Giulivi C , Tsolis RM , Revzin A , Lebrilla CB , Bäumler AJ . 2017. Microbiota-activated PPAR-γ-signaling inhibits Dysbiotic Enterobacteriaceae expansion. Science 357:570–575. doi:10.1126/science.aam9949 28798125 PMC5642957

[9] Schulthess J , Pandey S , Capitani M , Rue-Albrecht KC , Arnold I , Franchini F , Chomka A , Ilott NE , Johnston DGW , Pires E , McCullagh J , Sansom SN , Arancibia-Cárcamo CV , Uhlig HH , Powrie F . 2019. The short chain fatty acid butyrate imprints an antimicrobial program in macrophages. Immunity 50:432–445. doi:10.1016/j.immuni.2018.12.018 30683619 PMC6382411

[10] Belkaid Y , Hand TW . 2014. Role of the microbiota in immunity and inflammation. Cell 157:121–141. doi:10.1016/j.cell.2014.03.011 24679531 PMC4056765

[11] Evans L , Rhodes A , Alhazzani W , Antonelli M , Coopersmith CM , French C , Machado FR , Mcintyre L , Ostermann M , Prescott HC , Schorr C , Simpson S , Wiersinga WJ , Alshamsi F , Angus DC , Arabi Y , Azevedo L , Beale R , Beilman G , Belley-Cote E , Burry L , Cecconi M , Centofanti J , Coz Yataco A , De Waele J , Dellinger RP , Doi K , Du B , Estenssoro E , Ferrer R , Gomersall C , Hodgson C , Møller MH , Iwashyna T , Jacob S , Kleinpell R , Klompas M , Koh Y , Kumar A , Kwizera A , Lobo S , Masur H , McGloughlin S , Mehta S , Mehta Y , Mer M , Nunnally M , Oczkowski S , Osborn T , Papathanassoglou E , Perner A , Puskarich M , Roberts J , Schweickert W , Seckel M , Sevransky J , Sprung CL , Welte T , Zimmerman J , Levy M . 2021. Surviving sepsis campaign: international guidelines for management of sepsis and septic shock 2021. Intensive Care Med 47:1181–1247. doi:10.1007/s00134-021-06506-y 34599691 PMC8486643

[12] Branch-Elliman W , O’Brien W , Strymish J , Itani K , Wyatt C , Gupta K . 2019. Association of duration and type of surgical prophylaxis with antimicrobial-associated adverse events. JAMA Surg 154:590–598. doi:10.1001/jamasurg.2019.0569 31017647 PMC6487902

[13] Bell BG , Schellevis F , Stobberingh E , Goossens H , Pringle M . 2014. A systematic review and meta-analysis of the effects of antibiotic consumption on antibiotic resistance. BMC Infect Dis 14:13. doi:10.1186/1471-2334-14-13 24405683 PMC3897982

[14] Rooney AM , Timberlake K , Brown KA , Bansal S , Tomlinson C , Lee K-S , Science M , Coburn B . 2020. Each additional day of antibiotics is associated with lower gut anaerobes in neonatal intensive care unit patients. Clin Infect Dis 70:2553–2560. doi:10.1093/cid/ciz698 31367771 PMC7286368

[15] Baunwall SMD , Lee MM , Eriksen MK , Mullish BH , Marchesi JR , Dahlerup JF , Hvas CL . 2020. Faecal Microbiota transplantation for recurrent Clostridioides difficile infection: an updated systematic review and meta-analysis. EClinicalMedicine 29–30:100642. doi:10.1016/j.eclinm.2020.100642 PMC7788438 33437951

[16] Ianiro G , Murri R , Sciumè GD , Impagnatiello M , Masucci L , Ford AC , Law GR , Tilg H , Sanguinetti M , Cauda R , Gasbarrini A , Fantoni M , Cammarota G . 2019. Incidence of bloodstream infections, length of hospital stay, and survival in patients with recurrent Clostridioides difficile infection treated with fecal microbiota transplantation or antibiotics: a prospective cohort study. Ann Intern Med 171:695–702. doi:10.7326/M18-3635 31683278

[17] Seekatz AM , Young VB . 2014. Clostridium difficile and the microbiota. J Clin Invest 124:4182–4189. doi:10.1172/JCI72336 25036699 PMC4191019

[18] Saha S , Tariq R , Tosh PK , Pardi DS , Khanna S . 2019. Faecal microbiota transplantation for eradicating carriage of multidrug-resistant organisms: a systematic review. Clin Microbiol Infect 25:958–963. doi:10.1016/j.cmi.2019.04.006 30986562

[19] Tamburini FB , Andermann TM , Tkachenko E , Senchyna F , Banaei N , Bhatt AS . 2018. Precision identification of diverse bloodstream pathogens in the gut microbiome. Nat Med 24:1809–1814. doi:10.1038/s41591-018-0202-8 30323331 PMC6289251

[20] Millan B , Park H , Hotte N , Mathieu O , Burguiere P , Tompkins TA , Kao D , Madsen KL . 2016. Fecal microbial transplants reduce antibiotic-resistant genes in patients with recurrent Clostridium difficile infection. Clin Infect Dis 62:1479–1486. doi:10.1093/cid/ciw185 27025836 PMC4885654

[21] Leung V , Vincent C , Edens TJ , Miller M , Manges AR . 2018. Antimicrobial resistance gene acquisition and depletion following fecal Microbiota transplantation for recurrent Clostridium difficile infection. Clin Infect Dis 66:456–457. doi:10.1093/cid/cix821 29020222 PMC5850035

[22] Langdon A , Schwartz DJ , Bulow C , Sun X , Hink T , Reske KA , Jones C , Burnham C-AD , Dubberke ER , Dantas G , CDC Prevention Epicenter Program . 2021. Microbiota restoration reduces antibiotic-resistant bacteria gut colonization in patients with recurrent Clostridioides difficile infection from the open-label PUNCH CD study. Genome Med 13:28. doi:10.1186/s13073-021-00843-9 33593430 PMC7888090

[23] Hallowell HA , Gao AL , Suez J . 2023. Double-edged sword: impact of fecal microbiome transplants on the gut resistome. Curr Opin Gastroenterol 39:16–22. doi:10.1097/MOG.0000000000000894 36504032

[24] DeFilipp Z , Bloom PP , Torres Soto M , Mansour MK , Sater MRA , Huntley MH , Turbett S , Chung RT , Chen Y-B , Hohmann EL . 2019. Drug-resistant E. coli bacteremia transmitted by fecal microbiota transplant. N Engl J Med 381:2043–2050. doi:10.1056/NEJMoa1910437 31665575

[25] Hota SS , McNamara I , Jin R , Kissoon M , Singh S , Poutanen SM . 2019. Challenges establishing a multi-purpose fecal microbiota transplantation stool donor program in Toronto, Canada. J Assoc Med Microbiol Infect Dis Can 4:218–226. doi:10.3138/jammi.2019-0003 36339288 PMC9612805

[26] Kao D , Wong K , Franz R , Cochrane K , Sherriff K , Chui L , Lloyd C , Roach B , Bai AD , Petrof EO , Allen-Vercoe E . 2021. The effect of a microbial ecosystem therapeutic (MET-2) on recurrent Clostridioides difficile infection: a phase 1, open-label, single-group trial. Lancet Gastroenterol Hepatol 6:282–291. doi:10.1016/S2468-1253(21)00007-8 33631102

[27] Feuerstadt P , Louie TJ , Lashner B , Wang EEL , Diao L , Bryant JA , Sims M , Kraft CS , Cohen SH , Berenson CS , Korman LY , Ford CB , Litcofsky KD , Lombardo M-J , Wortman JR , Wu H , Auniņš JG , McChalicher CWJ , Winkler JA , McGovern BH , Trucksis M , Henn MR , von Moltke L . 2022. SER-109, an oral microbiome therapy for recurrent Clostridioides difficile infection. N Engl J Med 386:220–229. doi:10.1056/NEJMoa2106516 35045228

[28] Dsouza M , Menon R , Crossette E , Bhattarai SK , Schneider J , Kim Y-G , Reddy S , Caballero S , Felix C , Cornacchione L , Hendrickson J , Watson AR , Minot SS , Greenfield N , Schopf L , Szabady R , Patarroyo J , Smith W , Harrison P , Kuijper EJ , Kelly CP , Olle B , Bobilev D , Silber JL , Bucci V , Roberts B , Faith J , Norman JM . 2022. Colonization of the live biotherapeutic product VE303 and modulation of the microbiota and metabolites in healthy volunteers. Cell Host Microbe 30:583–598. doi:10.1016/j.chom.2022.03.016 35421353

[29] Petrof EO , Gloor GB , Vanner SJ , Weese SJ , Carter D , Daigneault MC , Brown EM , Schroeter K , Allen-Vercoe E . 2013. Stool substitute transplant therapy for the eradication of Clostridium difficile infection: 'RePOOPulating' the gut. Microbiome 1:3. doi:10.1186/2049-2618-1-3 24467987 PMC3869191

[30] Louie T , Golan Y , Khanna S , Bobilev D , Erpelding N , Fratazzi C , Carini M , Menon R , Ruisi M , Norman JM , Faith JJ , Olle B , Li M , Silber JL , Pardi DS . 2023. VE303, a defined bacterial consortium, for prevention of recurrent Clostridioides difficile infection a randomized clinical trial. JAMA 329:1356–1366. doi:10.1001/jama.2023.4314 37060545 PMC10105904

[31] Abujamel T , Cadnum JL , Jury LA , Sunkesula VCK , Kundrapu S , Jump RL , Stintzi AC , Donskey CJ . 2013. Defining the vulnerable period for re-establishment of Clostridium difficile colonization after treatment of C. difficile infection with oral vancomycin or metronidazole. PLoS One 8:e76269. doi:10.1371/journal.pone.0076269 24098459 PMC3788714

[32] Zuo T , Wong SH , Lam K , Lui R , Cheung K , Tang W , Ching JYL , Chan PKS , Chan MCW , Wu JCY , Chan FKL , Yu J , Sung JJY , Ng SC . 2018. Bacteriophage transfer during faecal microbiota transplantation in Clostridium difficile infection is associated with treatment outcome. Gut 67:634–643. doi:10.1136/gutjnl-2017-313952 28539351 PMC5868238

[33] Bonten MJ , Willems R , Weinstein RA . 2001. Vancomycin-resistant enterococci: why are they here, and where do they come from. Lancet Infect Dis 1:314–325. doi:10.1016/S1473-3099(01)00145-1 11871804

[34] Zhang A-N , Gaston JM , Dai CL , Zhao S , Poyet M , Groussin M , Yin X , Li L-G , van Loosdrecht MCM , Topp E , Gillings MR , Hanage WP , Tiedje JM , Moniz K , Alm EJ , Zhang T . 2021. An omics-based framework for assessing the health risk of antimicrobial resistance genes. Nat Commun 12:4765. doi:10.1038/s41467-021-25096-3 34362925 PMC8346589

[35] Kwak S , Choi J , Hink T , Reske KA , Blount K , Jones C , Bost MH , Sun X , Burnham C-AD , Dubberke ER , Dantas G , CDC Prevention Epicenter Program . 2020. Impact of investigational microbiota therapeutic RBX2660 on the gut microbiome and resistome revealed by a placebo-controlled clinical trial. Microbiome 8:125. doi:10.1186/s40168-020-00907-9 32862830 PMC7457799

[36] Jernberg C , Löfmark S , Edlund C , Jansson JK . 2007. Long-term ecological impacts of antibiotic administration on the human intestinal microbiota. ISME J 1:56–66. doi:10.1038/ismej.2007.3 18043614

[37] Rooney AM , Raphenya AR , Melano RG , Seah C , Yee NR , MacFadden DR , McArthur AG , Schneeberger PHH , Coburn B , Langelier CR , X-Z L . 2022. Performance characteristics of next-generation sequencing for the detection of antimicrobial resistance determinants in Escherichia coli genomes and metagenomes. mSystems 7:e0002222. doi:10.1128/msystems.00022-22 35642524 PMC9238399

[38] Shimasaki T , Seekatz A , Bassis C , Rhee Y , Yelin RD , Fogg L , Dangana T , Cisneros EC , Weinstein RA , Okamoto K , Lolans K , Schoeny M , Lin MY , Moore NM , Young VB , Hayden MK , Centers for Disease Control and Prevention Epicenters Program . 2019. Increased relative abundance of Klebsiella pneumoniae Carbapenemase-producing Klebsiella pneumoniae within the gut microbiota is associated with risk of bloodstream infection in long-term acute care hospital patients. Clin Infect Dis 68:2053–2059. doi:10.1093/cid/ciy796 30239622 PMC6541703

[39] Caporaso JG , Lauber CL , Walters WA , Berg-Lyons D , Huntley J , Fierer N , Owens SM , Betley J , Fraser L , Bauer M , Gormley N , Gilbert JA , Smith G , Knight R . 2012. Ultra-high-throughput microbial community analysis on the illumina HiSeq and MiSeq platforms. ISME J 6:1621–1624. doi:10.1038/ismej.2012.8 22402401 PMC3400413

[40] Andrews S 2010. FastQC: a quality control tool for high throughput sequence data. https://www.bioinformatics.babraham.ac.uk/projects/fastqc/

[41] Bolger AM , Lohse M , Usadel B . 2014. Trimmomatic: a flexible Trimmer for illumina sequence data. Bioinformatics 30:2114–2120. doi:10.1093/bioinformatics/btu170 24695404 PMC4103590

[42] Huttenhower C 2022. KneadData. https://huttenhower.sph.harvard.edu/kneaddata/

[43] Truong DT , Franzosa EA , Tickle TL , Scholz M , Weingart G , Pasolli E , Tett A , Huttenhower C , Segata N . 2015. MetaPhlAn2 for enhanced metagenomic taxonomic profiling. Nat Methods 12:902–903. doi:10.1038/nmeth.3589 26418763

[44] Nurk S , Meleshko D , Korobeynikov A , Pevzner PA . 2017. MetaSPAdes: a new versatile metagenomic assembler. Genome Res 27:824–834. doi:10.1101/gr.213959.116 28298430 PMC5411777

[45] Alcock BP , Raphenya AR , Lau TTY , Tsang KK , Bouchard M , Edalatmand A , Huynh W , Nguyen A-LV , Cheng AA , Liu S , Min SY , Miroshnichenko A , Tran H-K , Werfalli RE , Nasir JA , Oloni M , Speicher DJ , Florescu A , Singh B , Faltyn M , Hernandez-Koutoucheva A , Sharma AN , Bordeleau E , Pawlowski AC , Zubyk HL , Dooley D , Griffiths E , Maguire F , Winsor GL , Beiko RG , Brinkman FSL , Hsiao WWL , Domselaar GV , McArthur AG . 2020. CARD 2020: antibiotic resistome surveillance with the comprehensive antibiotic resistance database. Nucleic Acids Res 48:D517–D525. doi:10.1093/nar/gkz935 31665441 PMC7145624

[46] Buchfink B , Xie C , Huson DH . 2015. Fast and sensitive protein alignment using DIAMOND. Nat Methods 12:59–60. doi:10.1038/nmeth.3176 25402007

[47] Edgar RC . 2010. Search and clustering orders of magnitude faster than BLAST. Bioinformatics 26:2460–2461. doi:10.1093/bioinformatics/btq461 20709691

[48] Edgar RC . 2013. UPARSE: highly accurate OTU sequences from microbial amplicon reads. Nat Methods 10:996–998. doi:10.1038/nmeth.2604 23955772

[49] Edgar RC . 2016. UNOISE2: improved error-correction for illumina 16S and ITS amplicon sequencing. Biorxiv. doi:10.1101/081257

[50] Rognes T , Flouri T , Nichols B , Quince C , Mahé F . 2016. VSEARCH: a Versatile open source tool for metagenomics. PeerJ 4:e2584. doi:10.7717/peerj.2584 27781170 PMC5075697

[51] Wang Q , Garrity GM , Tiedje JM , Cole JR . 2007. Nave bayesian classifier for rapid assignment of rRNA sequences into the new bacterial Taxonomy. Appl Environ Microbiol 73:5261–5267. doi:10.1128/AEM.00062-07 17586664 PMC1950982

[52] Bolyen E , Rideout JR , Dillon MR , Bokulich NA , Abnet CC , Al-Ghalith GA , Alexander H , Alm EJ , Arumugam M , Asnicar F , Bai Y , Bisanz JE , Bittinger K , Brejnrod A , Brislawn CJ , Brown CT , Callahan BJ , Caraballo-Rodríguez AM , Chase J , Cope EK , Da Silva R , Diener C , Dorrestein PC , Douglas GM , Durall DM , Duvallet C , Edwardson CF , Ernst M , Estaki M , Fouquier J , Gauglitz JM , Gibbons SM , Gibson DL , Gonzalez A , Gorlick K , Guo J , Hillmann B , Holmes S , Holste H , Huttenhower C , Huttley GA , Janssen S , Jarmusch AK , Jiang L , Kaehler BD , Kang KB , Keefe CR , Keim P , Kelley ST , Knights D , Koester I , Kosciolek T , Kreps J , Langille MGI , Lee J , Ley R , Liu Y-X , Loftfield E , Lozupone C , Maher M , Marotz C , Martin BD , McDonald D , McIver LJ , Melnik AV , Metcalf JL , Morgan SC , Morton JT , Naimey AT , Navas-Molina JA , Nothias LF , Orchanian SB , Pearson T , Peoples SL , Petras D , Preuss ML , Pruesse E , Rasmussen LB , Rivers A , Robeson MS , Rosenthal P , Segata N , Shaffer M , Shiffer A , Sinha R , Song SJ , Spear JR , Swafford AD , Thompson LR , Torres PJ , Trinh P , Tripathi A , Turnbaugh PJ , Ul-Hasan S , van der Hooft JJJ , Vargas F , Vázquez-Baeza Y , Vogtmann E , von Hippel M , Walters W , Wan Y , Wang M , Warren J , Weber KC , Williamson CHD , Willis AD , Xu ZZ , Zaneveld JR , Zhang Y , Zhu Q , Knight R , Caporaso JG . 2019. Author correction: reproducible, interactive, scalable and extensible microbiome data science using QIIME 2. Nat Biotechnol 37:1091. doi:10.1038/s41587-019-0252-6 PMC7015180 31341288

[53] Bacchetti De Gregoris T , Aldred N , Clare AS , Burgess JG . 2011. Improvement of phylum-and class-specific primers for real-time PCR quantification of bacterial taxa. J Microbiol Methods 86:351–356. doi:10.1016/j.mimet.2011.06.010 21704084

[54] Brenner DJ , Krieg NR , Staley JT , Garrity GM , Boone DR , Vos P , Goodfellow M , Rainey FA , Schleifer K-H . 2005. The Proteobacteria part B, the Gammaproteobacteria. 2nd ed. Vol. 2. Springer-Verlag. https://www.springer.com/gp/book/9780387950402.

[55] Brenner DJ , Krieg N , Staley JT . 2005. The *Proteobacteria* part C, the Alpha-, Beta-, Delta-, and Epsilonproteobacteria. In Bergey’s Manual of systematic Bacteriology, 2nd ed. Vol. 2. Springer-Verlag, New York. doi:10.1007/0-387-29298-5

[56] Vos P , Garrity MG , Jones D , Krieg R , Ludwig W , Rainey A , Schleifer KH , Whitman B , The Firmicutes . 2009. Bergey’s Manual of systematic Bacteriology. 2nd ed. Vol. 3. Springer-Verlag, New York.

[57] Krieg NR , Staley JT , Brown DR , Hedlund BP , Paster BJ , Ludwig W , Whitman WB . 2010. The *Bacteroidetes*, *Spirochaetes*, *Tenericutes* (*Mollicutes*), *Acidobacteria*, *Fibrobacteres*, *Fusobacteria*, *Dictyoglomi*, *Gemmatimonadetes*, *Lentisphaerae*, *Verrucomicrobia*, *Chlamydiae*, and *Planctomycetes*. In Bergey’s Manual of systematic Bacteriology, 2nd ed. Vol. 4. Springer-Verlag, New York.

[58] Whitman WB , Goodfellow M , Kämpfer P , Busse H-J , Trujillo ME , Suzuki K-I . 2012. The *Actinobacteria*. In Bergey’s Manual of systematic Bacteriology, 2nd ed. Vol. 5. Springer-Verlag, New York.

