# Supplementary material for: A microbial consortium alters intestinal Pseudomonadota and antimicrobial resistance genes in individuals with recurrent Clostridioides difficile infection
Source: mBio. 2023 Jul 5;14(4):e03482-22. doi: 10.1128/mbio.03482-22 (PMC10506460; doi:10.1128/mbio.03482-22)
Supplement: Supplemental Figures — Figures S1 to S8. [file mbio.03482-22-s0001.docx]

**
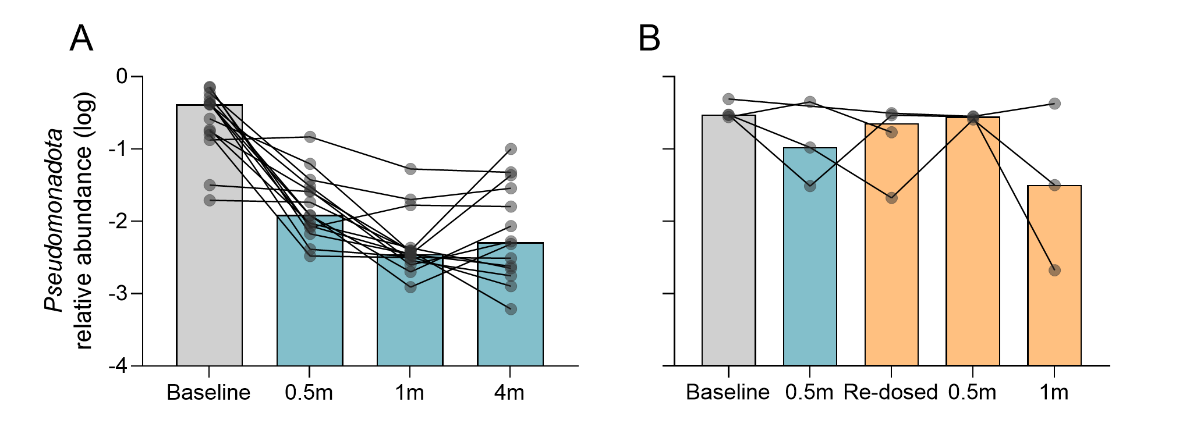
Supplementary Figures**

**Supplementary Figure 1. A-B,** *Pseudomonadota* relative abundance measured from 16S rRNA sequencing data generated previously from all participants (n = 19) of the initial MET-2 trial (26). **A,** *Pseudomonadota* measured at baseline (prior to MET-2) and at follow-up (post-MET-2) in months (m), in individuals (n = 15) who received a single course of MET-2. **B**, *Pseudomonadota* measured at baseline (prior to MET-2) as well as during re-dosing (Re-dosed) and at follow-up timepoints (post-MET-2) in months (m), in individuals (n = 4) who were re-dosed with MET-2. Values are log-transformed, where dots represent individual patients with lines connecting the same patients measured at different time points. Medians are plotted.


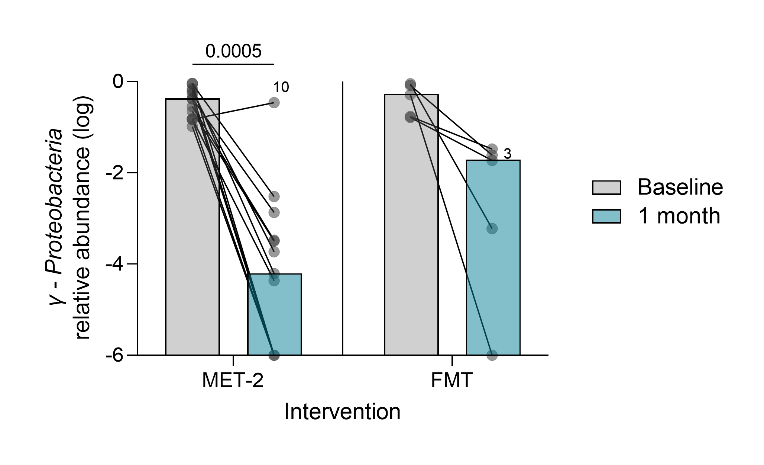


**Supplementary Figure 2.** *γ-Proteobacteria* relative abundance between baseline and 1-month post-intervention. Values are log-transformed, where dots represent individual patients with lines connecting the same patients measured at different time points. Participant 10 and participant 3 are highlighted as individuals who failed initial MET-2 or FMT therapy, respectively. Medians are plotted with the p-value displayed above the MET-2 interventional group. Pairwise analysis performed using Wilcoxon matched-pairs signed rank test.


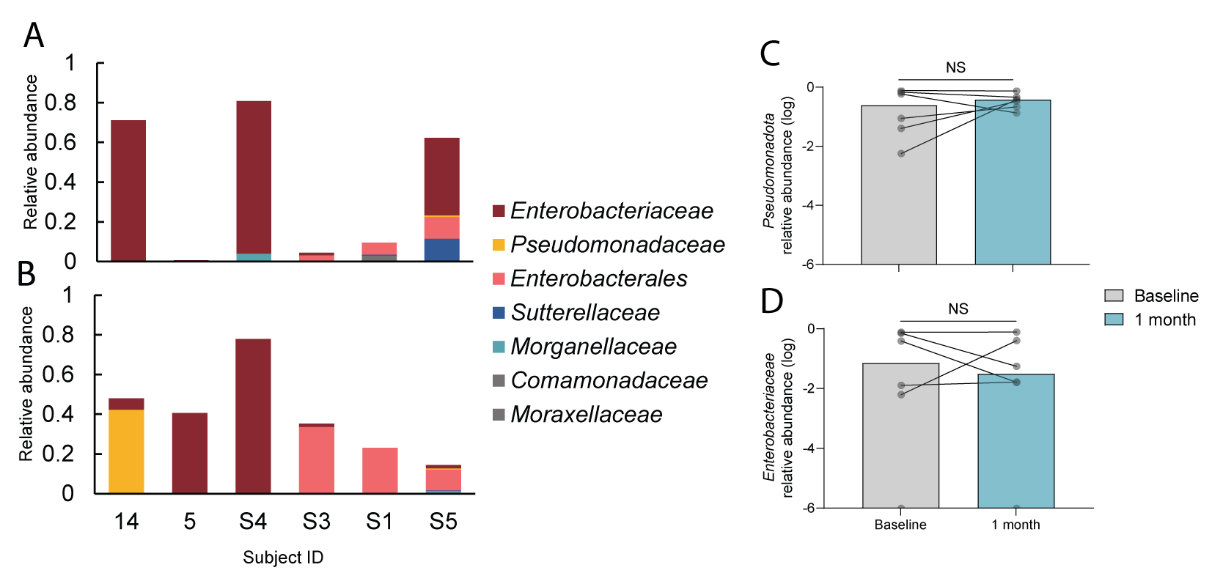


**Supplementary Figure 3.** Histograms of the *Pseudomonadota* relative abundances at baseline (**A**) and 1-month (**B**) post-vancomycin therapy in patients with CDI (n = 6) classified to the family-level. Taxa representing <5% relative abundance are coloured grey. **C**, *Pseudomonadota* and (**D**) *Enterobacteriaceae* relative abundances between baseline and 1-month post-intervention. **C-D**, Values are log-transformed, where dots represent individual patients with lines connecting the same patients measured at different time points. Medians are plotted. Pairwise analysis performed using Wilcoxon matched-pairs signed rank test. P-values were not significant (NS).


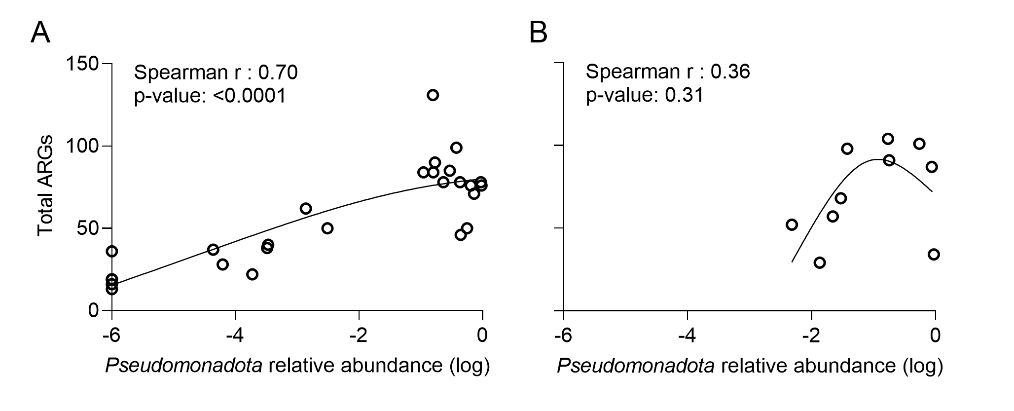


**Supplementary Figure 4. A-B,** The relationship between the number of ARGs and *Pseudomonadota* relative abundance for individuals who received MET-2 (**A**) and for individuals who received FMT (**B**). **A-B,** Splines are plotted to demonstrate trends. Spearman’s correlation was calculated to measure the relationship between ARGs and *Pseudomonadota* log-transformed relative abundance, Spearman’s Rho and p-values are plotted. Each dot represents an individual with the baseline and 1-month time points included.


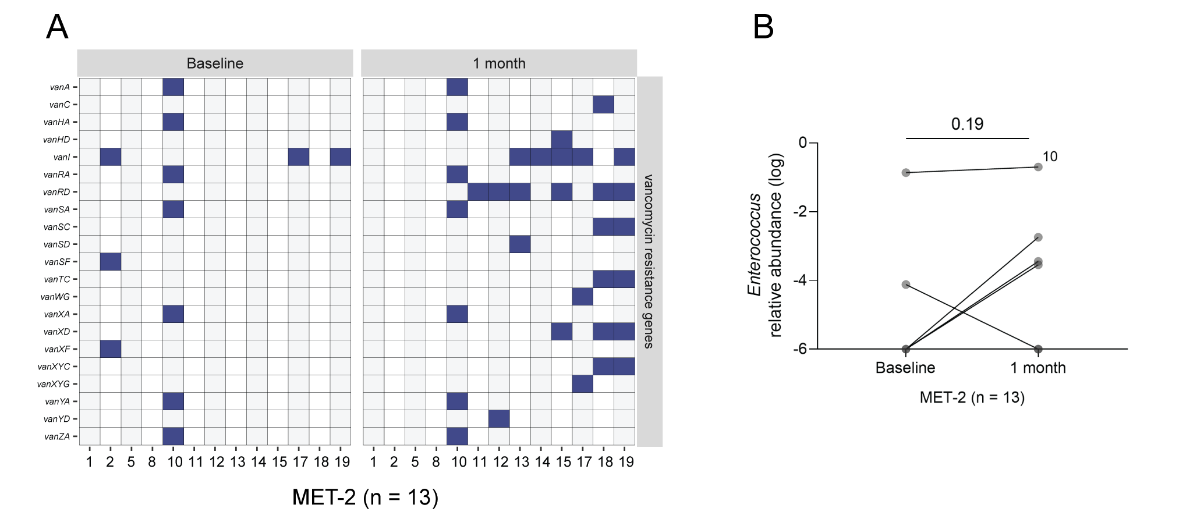


**Supplementary Figure 5. A,** Vancomycin resistance genes detected in stool samples at baseline and 1-month post MET-2 administration. Blue squares represent presence of a vancomycin resistance gene. **B**, *Enterococcus* relative abundance (log-transformed) in participants who received MET-2 (n = 13) between baseline and 1-month post-intervention. Dots represent individual patients with lines connecting the same patients measured at different time points. Participant 10 is highlighted as an individual who failed initial MET-2 therapy. Medians are plotted with the p-value displayed above the time points. Pairwise analysis performed using Wilcoxon matched-pairs signed rank test.


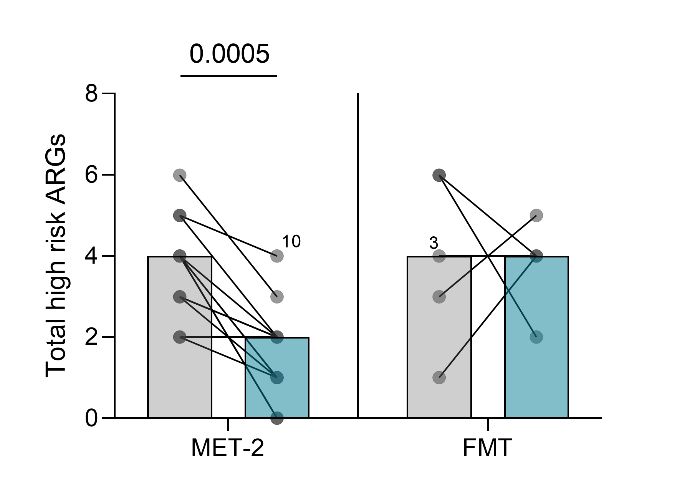


**Supplementary Figure 6.** Total high risk antimicrobial resistance genes (ARGs) between baseline and 1-month post-intervention. Dots represent individual patients with lines connecting the same patients measured at different time points. Participant 10 and participant 3 are highlighted as individuals who failed initial MET-2 or FMT therapy, respectively. Medians are plotted with the p-value displayed above the MET-2 interventional group. Pairwise analysis performed using Wilcoxon matched-pairs signed rank test.


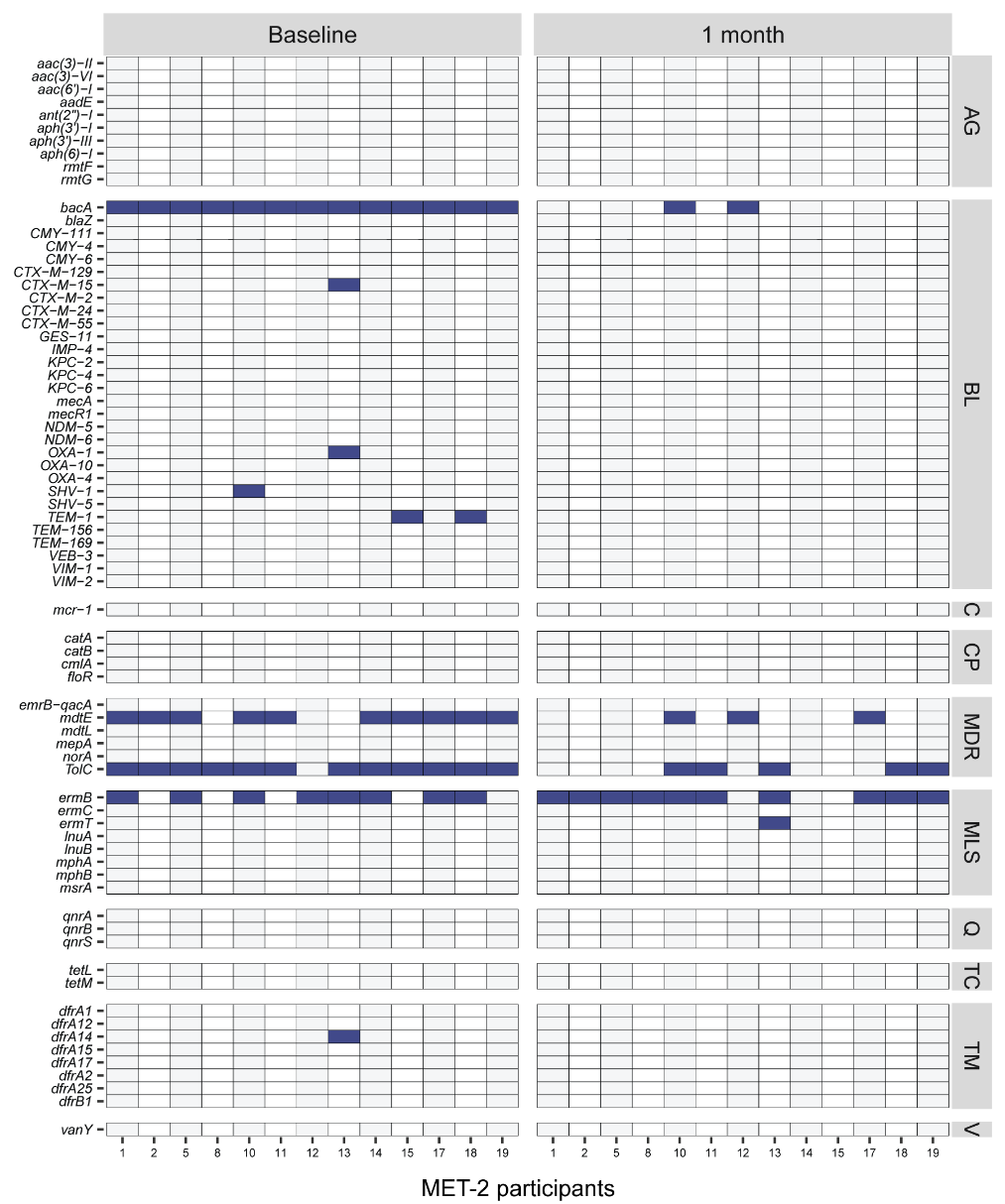


**Supplementary Figure 7.** High risk antimicrobial resistance genes (ARGs) detected in stool samples at baseline and 1-month post MET-2 administration. Blue squares represent presence of a high risk ARG. ARGs are categorized by antibiotics they confer resistance to, including aminoglycosides (AG), beta-lactams (BL), colistin (C), chloramphenicol (CP), multidrug resistance (MDR), macrolides, lincosamides, streptogramines (MLS), quinolones (Q), tetracyclines (TC), trimethoprim (TM), and vancomycin (V).


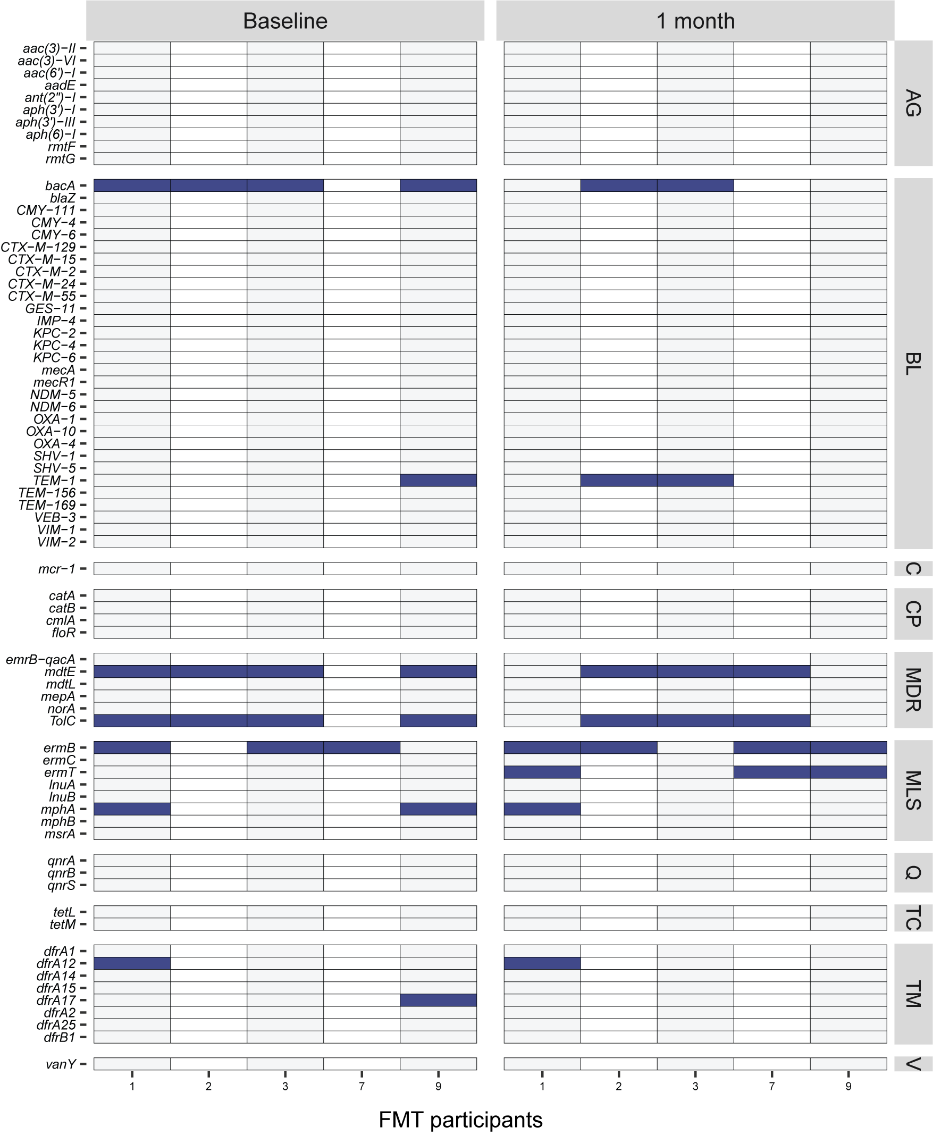


**Supplementary Figure 8.** High risk antimicrobial resistance genes (ARGs) detected in stool samples at baseline and 1-month post FMT administration. Blue squares represent presence of a high risk ARG. ARGs are categorized by antibiotics they confer resistance to, including aminoglycosides (AG), beta-lactams (BL), colistin (C), chloramphenicol (CP), multidrug resistance (MDR), macrolides, lincosamides, streptogramines (MLS), quinolones (Q), tetracyclines (TC), trimethoprim (TM), and vancomycin (V).
