# Supplementary material for: A microbial consortium alters intestinal Pseudomonadota and antimicrobial resistance genes in individuals with recurrent Clostridioides difficile infection
Source: mBio. 2023 Jul 5;14(4):e03482-22. doi: 10.1128/mbio.03482-22 (PMC10506460; doi:10.1128/mbio.03482-22)
Supplement: Table S1 — Participant characteristics. [file mbio.03482-22-s0003.docx]

**Supplementary Table 1**. Patient characteristics

| **Characteristic** |  | **MET-2 (n= 15)** |  | **FMT (n = 5)** |
| --- | --- | --- | --- | --- |
| Female Sex, n (%) |  | 10 (67) |  | 5 (100) |
| Age,^a^ years (range) |  | 65 (49 – 90) |  | 67 (50 – 95) |
| CDI recurrence post-intervention, n (%) |  | 1 (6.7) |  | 1 (20) |
| Vancomycin suppression,^b^ n (%) |  | 15 (100) |  | 4 (80) |
